# Specialists’ Dual Practice within Public Hospital Setting: Evidence from Malaysia

**DOI:** 10.3390/healthcare10102097

**Published:** 2022-10-20

**Authors:** Malindawati Mohd Fadzil, Sharifa Ezat Wan Puteh, Azimatun Noor Aizuddin, Zafar Ahmed

**Affiliations:** 1 Department of Community Medicine, Faculty of Medicine, Universiti Kebangsaan Malaysia Medical Centre, Cheras, Kuala Lumpur 56000, Malaysia; 2 Medical Development Division, Ministry of Health Malaysia, Precinct 1, Putrajaya 62590, Malaysia; 3 Department of Social Work, Education and Community Wellbeing, Faculty of Health and Life Sciences, Northumbria University, Newcastle-upon-Tyne NE1 8SG, UK; 4 Department of Community Medicine and Public Health, Faculty of Medicine, University Malaysia Sarawak, Kota Samarahan 94300, Malaysia

**Keywords:** dual practice within public hospital, dual practice, public-on-private, full paying patient, private care, public and private healthcare sector, commercial wing, clinical specialist, retention

## Abstract

In line with the commitment of the Malaysian government and Ministry of Health to prevent the brain drain of specialists from public hospitals, they have been permitted to perform dual practice within the public hospital setting (DPH) since 2007. DPH allowed them to hold jobs in both public and private practices within the same public hospitals that they are affiliated to, permitting these specialists to treat public and private patients. Nevertheless, the information regarding DPH in Southeast Asia region is still limited. This narrative review provides insight into the implementation of DPH in Malaysia. It highlights that DPH has been well-governed and regulated by the MOH while serving as a means to retain specialists in the public healthcare system by providing them with opportunities to obtain additional income. Such a policy has also reduced the financial burden of the government in subsidizing healthcare. However, as in other countries with similar policies, multiple challenges have arisen from the implementation of DPH in Malaysia despite its positive achievements and potentials. This paper concludes that proactive governance, monitoring, and regulation are key to ensure the success of DPH.

## 1. Introduction

Globally, the retention of specialists in the public healthcare sector remains a major problem. The exodus of specialists from the government sector to the private sector is considered perilous to the public health sector since their skills and expertise are highly demanded to perform complex procedures to ensure the best treatment outcomes for patients [1]. They play an essential role in training the junior specialists by passing down their skills and knowledge to ensure sustainability in the provision of a high-quality, accessible, and equitable healthcare system [2].

Allowing specialists to take up dual practice is one of the common policy interventions to overcome attrition in low- and high-income countries [3]. Dual practice enables healthcare professionals to serve in private health settings without quitting the public sector [4,5,6]. Such a policy has been long adopted in many countries with a two-tier health system including Malaysia [5]. Other terms used to describe dual practice included ‘public-on-private’, ‘moonlighting’, or ‘multiple job holding’. However, the existing literature generally underlines the negative impacts of dual practice on the public healthcare system [6,7,8]. McPake et al. [9] highlighted the multifactorial and contextual differences on the impact of dual practice across countries, including regulatory environments and opportunities for such practice, and levels of demand for public and private healthcare services. Published studies reported on the different consequences of dual practice and the regulatory responses between high- and low-income countries [6,10,11].

It is crucial to distinguish the different types of dual practice to appreciate different arrangements implemented in numerous settings. In general, dual practice can be classified based on the locations of healthcare professionals attending to private patients as follows: (i) ‘outside’: in a separate private setting; (ii) ‘beside’: in a private ward or clinic physically connected with a public facility but run as a private business; (iii) ‘within’: in the same public facility but outside public service operating hours at extra fees, or (iv) ‘integrated’: in the same public facility with informal payment and no time restrictions [9,12]. In this context, ‘private patient’ refers to a patient who chooses and pays for their doctor and medical care, rather than using subsidized healthcare or the care provided for free by the government. The selection of dual practice type adopted in a country is closely linked to healthcare system governance and market demand for specialist services [12], even though the ‘outside’ type is the most discussed in the existing literature.

Full-time public servants who are solely dedicated to the public sector are no longer the norm. Dual practice is not limited to high-income countries and has also been widely adopted in low- and middle-income countries (LMIC) [4,10,11,13]. Dual practice within public hospitals (DPH), has been set up in developed and developing countries alike. The implementation of this model has been reported in Austria, Ireland, Italy, Australia, United Kingdom, France, and Germany [8,10,14,15], Indonesia [5], Vietnam [16], Ethiopia [17,18], and Uganda [19].

Multiple reasons for specialists taking up dual practice have been identified. According to Ferrinho et al. [4], the extent of specialist engagement in dual practice and their reasons are contextual in nature, even though additional incomes was often cited as the key motivation [10,17,20]. Ferrinho et al. [4] also highlighted dual practice as individual coping strategies against unrealistically low salaries. Socha and Bech [6] highlighted the possibility of different motivating factors for specialists who undertook dual practice in low- and high-income countries while García-Prado and González [10] discussed evidence to relate dual practice decisions with multiple factors such as working hour restrictions, job complementarities, as well as professional, institutional, and personal issues. Hipgrave and Hort [5] suggested that among non-financial reasons for dual practice in higher income countries are extra training to gain new skills and experience from private practice for self-improvement, to boost prestige, peer approval, and larger professional contact [10].

Both positive and negative consequences of DPH have been reported in the existing literature [5,6,14]. On a positive note, DPH enhanced the employer attractiveness of the public sector [14,21,22]. Such a policy was found to improve the retention of healthcare professionals in the public sector which successfully improved access to quality service delivery [17]. At the same time, DPH was also shown to generate additional income for public hospitals [14,21,22,23,24] and enhanced patient choice for treatment preference [14,23,25]. On the contrary, DPH has also been linked to negative consequences in terms of the service provision of public hospitals. Conceptually, DPH can lead to diminished healthcare responsiveness, distorted use of physical resources, and additional strain on the public hospital administration [14]. Consequently, it can lead to a conflict of interest, competition for specialists’ time and hospital resources, and limited access for non-private patients [4,5,14,24]. Worse still, it may also erode the public’s trust in the public healthcare system [14,23], especially if DPH is poorly regulated and managed. Within this context, Mueller and Socha-Dietrich [14] concluded that the net implications of DPH are ambiguous and are highly dependent on its governance.

In general, there is no single best method in the management of dual practice, with different strategies being adopted by governments [26]. In some countries, the government approach was to ban dual practice; for example, in China and Canada [10]. Prohibition as a means of controlling dual practice was deemed as highly ineffective, often resulting in an undesirable loss of valuable healthcare professionals [4,11,14]. In some countries such as Austria and the United Kingdom, dual practice is allowed but regulations are put in place to prevent or to alleviate the potential adverse consequences [11,13]. On the other extreme, dual practice without any restriction or regulation is generally not recommended because it will lead to various negative consequences [5].

Interestingly, the characteristic of DPH is viewed as one of the better option to facilitate the regulation of dual practice as it could be more easily controlled and governed [5,6,13,27]. To allay the concerns about conflicts of interest and competing priorities, some countries set a maximum amount of specialists’ earnings from DPH [13]. For example, public hospitals in France also shared a portion of the specialists’ earnings from DPH [14]. While previously, the National Health Service (NHS) in the UK limited the earnings from DPH to 10% of specialists’ incomes [14]. As González and Macho-Stadler [11] opined that limiting the activities of healthcare professionals rather than their incomes is likely to be more effective in regulating DPH, the NHS lifted the restriction in 2003 but emphasized specialists must ensure that their private practice does not result in a detrimental effect on NHS patients or services, nor diminish the public resources that are available for the NHS [28]. The plan to mandate income declarations was also cancelled in 2017 [14,29]. Apart from limiting the time devoted to DPH to less than 20% of worktime, France set a limit to the volume of services delivered [14]. DPH was also limited to senior physicians in some countries, such as Ireland [14], Kenya, and Zambia [8]. The types of enforcement are crucial as the arrangement of DPH has a larger implication in terms of higher risks of public resource mismanagement and conflict of interest [5,13,14].

In Malaysia, public specialists are allowed to perform both the ‘outside’ (locum at private hospitals) and ‘within’ types of dual practice [Full Paying Patient (FPP) Service]. Under the FPP, patients are treated by their specialists of choice in first-class wards and charged at full rate without receiving any government subsidies for the treatment [30]. Although FPP has long been offered since 2007, the information regarding its implementation is still limited. Therefore, this narrative review aimed to fill the gap by summarizing the evidence for the implementation of DPH in Malaysia, focusing on specialists serving in the public healthcare sector. We describe the extent of DPH, the motivations behind its implementation, the consequences of the practice, the service regulatory measures by the Ministry of Health (MOH) Malaysia, and the main challenges of this type of dual practice.

### Background of DPH in Malaysia

Malaysia is an upper-middle-income country with a two-tier health system. The public healthcare sector is funded predominantly through general taxation in co-existence with a market-driven private health sector that consists of mostly physician-owned clinics and hospitals [31]. All citizens in the country are entitled to a range of subsidized healthcare services, including both inpatient and outpatient care in the public system with nominal user charges [32]. The total expenditure on health (TEH) in Malaysia between 1997 to 2019 ranged from 3.04% to 4.30% of the Gross Domestic Product (GDP). In 2019, the TEH was 4.3% of GDP, 52% of which was contributed by the public sector [33]. As high as 99% of the health expenditure in the public sector was subsidized by the government and patients only co-pay 1–2% of the total cost [34]. More than half of the health expenditure in the public sector (55%) in 2019 was allocated to the wages for the employees of the MOH [35].

The 67th World Health Assembly adopted the Global Strategy on Human Resources for Health: Workforce 2030 with the aim to ensure universal availability, accessibility, acceptability, coverage, and quality of the health workforce through adequate investment to strengthen health systems and implementation of effective policies [36]. To pursue universal health coverage, all countries need adequate qualified medical specialists of multiple disciplines [9]. Nonetheless, the density of various clinical specialists in Malaysia is still relatively low (3.23 specialists per 10,000 populations). The selected member states of the Organization for Economic Co-operation and Development (OECD), for example, recorded an average density as high as 22.4 specialists per 10,000 populations [31].

Approximately 60% of the specialists in Malaysia serve in public hospitals [31]. They are responsible for the care provided to 70% of the inpatients in Malaysia, not to mention their role in training junior doctors [37]. The bed capacity in public hospitals is also nearly three times larger than that of private hospitals [38]. However, the rapid expansion of the private health sector catalyzes the loss of medical specialists in public hospitals. According to Amir and Ezat [2], 6.4% of MOH specialists left the public healthcare sector in 2000. They attributed the leaving of specialists from the public healthcare sector to job dissatisfaction due to suboptimal work environment, the lack of career paths, ineffective management and low salaries.

The Malaysian government has introduced multiple strategies to retain specialists in the public health sector, including offering financial incentives. Financial incentives play an important role in influencing individuals’ choice of workplace and have always been applied to address workforce shortage in healthcare worldwide [3,39]. While public sector health workforce under MOH receive fixed monthly salaries according to a salary scale with bands that vary by professional classification and grade, their counterparts in the private sector are mainly remunerated through fee-for-service payments [32,40]. On top of the fixed monthly salaries, they are entitled to specific allowances which make up approximately 20% and 50% of the total gross salary of non-specialists and specialists, respectively [40]. Currently, senior specialists in public hospitals could earn up to RM 14,000 excluding allowances and can make up to approximately RM 21,000 per month including allowances [41,42]. It has been claimed that private hospitals are often able to provide a 10-times-higher pay [43]. Regarding working hours, medical doctors in Malaysia generally need to work for at least 75 h weekly [44]. To reduce the attrition of specialists, time-based promotion and the permission to practice in private healthcare settings (locum) during out-of-office hours were also introduced [40]. Despite these initiatives, 30% of medical specialists in Malaysia had a tendency to quit the public sector within three years due to their discontentment with salaries, benefits and allowances, and location and facilities of workplaces being the significant contributing factors [45].

In many LMICs, seeking additional incomes by taking on fee-for-service private patients is common among healthcare workers [11,17]. Resorting to dual practice to meet their financial needs implied the inability of health ministries to ensure sufficient salaries [4]. In view of this, it may be more feasible and practical for the public sector to keep their skilled health workers at a low budgetary cost by allowing them to perform dual practice [10,46]. ‘FFP Service’ emerged against such a background in Malaysia [47].

According to Rahim and Mwanri [39], there is a paucity of evidence, particularly in the Malaysian context, on the long-term retention of health professionals. On top of that, despite the rapid expansion of mixed healthcare systems in the South and East Asia region, Hipgrave and Hort [5] highlighted the scanty evidence of dual practice in Southeast Asia. More research is required to inform health policies related to the recruitment and retention of healthcare professionals in the public sector. Therefore, this paper specifically discussed the various aspects of the FPP Service implementation as DPH in the Malaysian public healthcare sector.

## 2. Method

This was a narrative review [48,49] focusing on medical specialists from public hospitals in Malaysia. The DPH implementation was summarized based upon the literature related to FPP Service. A public hospital is one that is government-owned and fully funded by the government. The keywords used in the search included ‘dual practice OR Full Paying Patient in public OR government hospitals in Malaysia’, ‘Full Paying Patient in Malaysia’, and ‘*Perkhidmatan Pesakit Bayar Penuh di Malaysia*’ (Malay). Literature searches were conducted using electronic databases (PubMed and Google Scholar) to identify peer-reviewed publications, including empirical evaluation, policy analysis, and reviews published from January 2016 to October 2021. Additional articles were also identified from bibliographies and grey literature. Websites of the MOH and research institutes were also explored. Due to a limited number of peer-reviewed articles available, this review was based mainly on grey literature, including official reports, press statements and guidelines. The documents meant for internal circulation in the MOH were not included in this review. Overall, the analysis in this review led to an overview of the operational policy, the scale of implementation, regulations, uptake, outcomes, and major challenges of the FPP Service in Malaysia.

### 2.1. Rationale of the FPP Service

The former Prime Minister of Malaysia pointed out that “The Government is not able to provide high remunerations for medical specialists. As such, the Government has agreed to set up private commercial wings in Government hospitals, to enable serving doctors to enjoy better remunerations and thereby, continue to serve with the Government. Through these measures, the Government also hopes to attract specialists who have left the service to return and serve in Government hospitals. In addition, this will enable those seeking better medical treatment to obtain such treatment at reasonable charges in Government hospitals. It will also enable our government hospitals to be promoted abroad, in line with the objective to encourage health tourism.” [47]. Such an initiative taken by the MOH was intended to retain medical specialists in the public sector while offering them additional incomes. It was also expected to reduce the financial burden of the government in subsidizing healthcare for patients who can afford to pay for their healthcare [30].

### 2.2. Concept of the FPP Service

The FPP Service was launched in 2007 [2,30]. It was first approved by the Malaysian Cabinet on the 13 October 2004. At national level, four committees, namely the Finance Committee, Organization and Human Resources Committee, Legal and Management Committee, and Service Scope Committee, were formed to formulate the policies, terms of reference, concepts, and the scope of the FPP service [50]. FPP Service manage by respective hospitals’ administration and regulated through the Fees (Medical) (Full Paying Patient) Order 2007 and the Guidelines for Implementation Fee Order (Medical) (Full Paying Patient) 2007 (Revision 2015). Within the same hospital setting, FPP service shares facilities, equipment and human resource [30,51]. The hospital-level FPP committee tasked to monitor the provision of the service and to ensure the smooth operation of FPP Service [2,30]. The FPP Service should not be viewed as the privatization or commercialization of public hospitals but as an alternative for patients who can afford to pay for healthcare [52]. It is noted that subsidized public healthcare service for the public shall continue as usual [34,52]. FPP patients can have access to specialists of preference, priority to seek treatment, and access to facilities in executive, or first-class ward or equivalent, depending on the resources and facilities available at the hospital [30,50,52,53,54]. In return, these patients are charged at a full rate without the usual healthcare subsidy by the government [30,34,50,52,55].

### 2.3. Implementation and Expansion of the FPP Service

The implementation of FPP Service started at only two tertiary hospitals, namely the Putrajaya Hospital and the Selayang Hospital, which are also known as the pioneer FPP hospitals [30,34]. The positive achievement based on the increasing trends of patient and specialist participation in FPP Service prompted its expansion [2]. The first phase of expansion started seven years later, involving eight more public hospitals [50,56,57]. In 2016, the planned second phase expansion to a further eight MOH hospitals was withheld to allow the strengthening of the governance and regulations of the FPP Service [50]. As of December 2020, FPP Service was available in ten public hospitals (Table 1) [50,52]. Moving forward, the MOH is envisioning the expansion of FPP Service in phases to more selected hospitals during 2021–2025 under the 12th Malaysia Plan [58].

### 2.4. Operational Policies and Guidelines of the FPP Service

The Guidelines for Implementation Fee Order (Medical) (Full Paying Patient) 2007 (Revision 2015) was developed to guide the FPP Service at the operational level. The guidelines cover the basic service operation from registration to payment, qualification of specialists to participate in the service, code of ethics, patient management and monitoring mechanism [30]. The scope of FPP Service widely ranges from outpatient, inpatient, and daycare services. The emergency care is excluded from FPP Service [30]. The inclusion criteria for patients who are keen to opt for this service include those who require elective procedures and those who are in non-emergency and uncomplicated conditions, low risk, and not expected to require intensive care after surgery [30].

Registration is made mandatory for medical specialists who would like to take up the FPP Service. The registration criteria include being a Malaysian citizen and have completed three years of specialist gazettement or as a subspecialty specialist [30]. All FPP specialists must adhere to the code of ethical conduct that outlines their responsibility for the treatment and care of patients. They need to ensure that their involvement in FPP Service does not affect their commitment to public patients and they must always give priority to providing the necessary treatment according to patients’ clinical needs [30]. Generally, the consultation of FPP patients can only be performed by FPP specialists after they have completed the consultation and treatment of public patients [30].

### 2.5. FPP Charges and Revenues

The types and rate of fees that can be charged on patients under the FPP Service are listed under the Fees (Medical) (Full Paying Patient Service) Order 2007 (FPP Fee Order) [34,50,52]. The FPP Fee Schedule was set based on the Medical Fee Order 1982, the Malaysian Medical Association (MMA) 4th edition Schedule of Fees, Thirteenth Schedule of the Private Healthcare Facilities and Services (Private Hospitals and Other Private Healthcare Facilities) Regulations 2006 fee schedule, and input from medical specialists and pharmacists [50]. Seven types of charges can be imposed on patients, including registration, consultations, investigations, procedures, treatment, hospitality, and miscellaneous charges such as health examination packages, medical reports, the use of consumables (medicines), and disposable items [30,53]. As for the methods of payment, FPP patients can opt to pay for the FPP Service out-of-pocket or be covered under their private insurance or employee benefits [30,52].

The FPP revenue collected by the hospital is divided into portions payable to the FPP specialists and the government [30,54,55] (Table 2). The government’s portion is not retained by the hospital or MOH, but is returned to the consolidated fund under the central treasury at Ministry of Finance [34,54].

### 2.6. Monitoring and Regulatory Measures for the FPP Service

The FPP Service is regulated for the number of specialist engagements with patients, amount of incomes, time spent, and the number of patients treated [30,32]. In general, FPP specialists’ engagements with FPP inpatients should not be more than two times per day except in certain circumstances such as on patient request or for emergency cases. The specialists’ monthly income generated from the FPP practice should not be three times higher than their salaries including allowances [30,54]. They are only allowed to consult or treat FPP patients after completing the necessary care for public patients. Any surgical procedure for FPP patients must be performed after working hours [30,32], except for emergencies and certain cases after consideration and permission from the Director of the Hospital [30]. For outpatient clinics, FPP specialists can treat no more than 30% of FPP patients out of the total outpatients at a particular time. This can only be carried out after they have finished the consultation session for public patients [30,32]. Generally, FPP Service has been well-governed and in compliance with all the rules set by MOH [50].

### 2.7. Motivations for FPP Service Uptake among Specialists

A qualitative study by Amir et al. [51] revealed reasons of taking up the FPP Service among specialists rather than joining private practice. Alongside job security and protection rendered by the government in the public sector, the FPP service allowed them to work in familiar surroundings with supports and guidance from senior specialists [51]. Apart from that, they are entitled with the privileges as civil servants, and their seniority and promotion prospect are not affected. Patients under the FPP Service were also less demanding patients compared to those seeking care from private hospitals. Moreover, the FPP Service gave them high job satisfaction and save them the inconvenience to travel to other hospitals for locum [51].

### 2.8. Outcomes of the FPP Service

A steady growth in the number of specialists registered for the FPP service has been observed [2,50,60]. As of December 2018, there were 360 FPP specialists from ten hospitals that registered for the service [60]. The percentage of specialists participating in the FPP Service from 2015 to 2017 increased from 21.6% to 29.6% (Supplementary Materials: Table S1) [50]. During the same period, the total number of specialists who resigned in ten FPP hospitals was 31.8% from the total of specialists who resigned from the MOH (Supplementary Materials: Table S2) [50]. This trend indicated that the specialists’ receptiveness towards FPP and its potential to retain specialists in the government sector [50]. Amir et al. [61] compared specialists’ resignation rates from 2014 to 2016 for Putrajaya Hospital and Selayang Hospital with MOH specialists’ resignation rate (Supplementary Materials: Table S3). In 2017 and 2018, FPP specialists increment rates were higher compared to both non-FPP MOH specialists and private healthcare specialists (Supplementary Materials: Table S4) [31,60]. However, Fun et al. [32] highlighted in their study that the actual extent of the contribution of FPP Service to the overall brain drain issue are probably under-explored.

There was a significant rise in FPP patients’ encounters from 2015 to 2019 following expansion of the FPP Service to another eight hospitals (Supplementary Materials: Figure S1) [60]. In 2017 to 2019, FPP patients’ encounters show a marked growth compared to non-FPP patients’ encounters at all MOH hospitals and patients’ encounters at private hospitals (Supplementary Materials: Table S5) [38,60,62,63,64,65] Though the rate of growth was significant for FPP patients, the percentage of FPP patients’ encounters accounted for only 0.13% to total of MOH patients’ encounters in 2019 (Supplementary Materials: Table S6) [38,60,62,63,64,65].

In a quantitative study conducted by Liyana et al. [56], FPP patients were compared with public patients in terms of their satisfaction levels towards medical care received. Self-administered questionnaires were used to measure the level of satisfaction incorporating seven subscales: general satisfaction, technical quality, interpersonal manner, communication, financial aspects, time spent with doctor, accessibility, and convenience. Both FPP patients and public patients were found to be satisfied with the medical care received at FPP hospitals. While FPP patients were more satisfied with the service accessibility and convenience, communication, and time spent with doctors, public patients were more satisfied with financial aspects of medical care. More importantly, the study reported that there was no significant difference in terms of the technical quality between FPP and public patients, therefore implying the same standard of care received by both groups [56].

Fun et al. [32] reported that FPP patients in one of the FPP hospital experienced seven times shorter waiting times for cataract surgery compared to public patients. Nevertheless, public patients’ waiting time for cataract surgery was better than that of Finland, Spain, the United Kingdom, Australia, and New Zealand. FPP patients were less affected by surgery rescheduling as compared to public patients even though most of the rescheduling was cited to be due to medical factors such as uncontrolled blood pressure. The optimization of public hospital resources such as operation theatre, equipment, utilities, and human resources were found to be more efficient with the FPP Service out-of-office hours operational time [32].

The FPP Service has generated a sizeable revenue and created an alternative source of income for the government since it was first implemented in 2007 [32]. The amount of FPP revenue continuously increased from 2012 to 2018 from RM 4.4 million to more than RM 22 million, respectively [34,50,60]. A study by Hairusnizan et al. [66] also showed same pattern of increased trend of revenue collection in Selayang and Putrajaya Hospitals. This has a positive impact for the Malaysia public healthcare sector since the revenue can lower the healthcare expenditure by offsetting healthcare subsidies among people who can afford to pay for medical treatment [32,34,50].

### 2.9. Challenges of the FPP Service

Following the public announcement for FPP Service implementation, there were major concerns from the public that this scheme would divert the attention of specialists from their public duty and compromise the quality of services received by public patients [67]. The MOH then reassured the public that the subsidized healthcare service would continue to be made available for the public and the FPP Service would not affect the existing services at any public hospitals [52,68].

The challenges faced by hospital authorities were briefly reported by Amir et al. [69]. In a study on providers’ perspective for FPP Service, increased workload with limited workforce in the FPP service delivery, coupled with limited beds, were the major barriers to the FPP service [69]. The National Audit Report [50] also highlighted the need for an IT system to ensure smooth operation of FPP Service in billing, revenue management, and remuneration of specialists. Another qualitative study by Amir et al. [51] on specialist’s perspective also highlighted the similar issue in resource management. In addition, the lack of transparency in the financial management, work process, guidelines and monitoring have raised concerns about the potential abuse of the health system [51]. Specialists would give more attention to patients who pay and perform unnecessary tests or procedures. Nevertheless, such finding needs to be verified by further investigations.

Several public specialists declined the opportunity to join FPP Service due to existing workloads, work exhaustion, or family commitment. Some felt comfortable with their current income or already had established work outside of the public hospital, while others were concerned with ethical issues and felt that the FPP guideline was unclear and that there was lack of administrative support from the hospital administration [51]. The same study also found that some physicians still resigned from the public sector despite the availability of FPP Service because they felt that the remuneration they received would still be less than what they could gain from the private hospitals. Others cited the burden of non-clinical workload, family commitment, as well as slow and limited job promotion in the public sector as their reasons for leaving [51].

Apart from that, the report by the National Audit Department [50] and a few studies highlighted some flaws in the existing FPP Fee Schedule, which does not include a wide range of commonly used items [51,57,69]. A cost and revenue analysis of the two pioneer FPP hospitals showed that even though the revenues showed an increasing trend between 2014 and 2016 [66], they were 16–52% lower than the estimated costs for both hospitals [57]. The revision of the fee schedule is a time-consuming and tedious process. Amendment to the fee schedule is subjected to the Fees Act 1951 and will involve complex legal processes that require approval from various levels [50]. It is understood that MOH is currently revising the fee schedule and the new schedule should be endorsed in the near future [50,58].

Lastly, FPP only remunerates the specialists but not nurses, medical assistants, radiographers, and other support staffs. They are only allowed to claim standard overtime payment for their out-of-office hour work [30]. This had led to discontentment and resulted in poor supports given by them for the FPP service delivery [69].

## 3. Discussion

To the best of our knowledge, this was the first review on the implementation of DPH in Malaysia, which covered the policy, practices and evaluation. It started with the information regarding the history of DPH in the country, followed by the extent of the implementation, governance, and regulation. We also discussed motivations for the uptake, achievement, and challenges of DPH based on the limited literature identified.

It is noted that the FPP service is only available in 10 out of 146 public hospitals in Malaysia to date. The implementation of FPP Service reveals that the distinctive realms between public and private healthcare are progressively blurry in this country. FPP Service is a reflection that the Malaysian mixed public-private healthcare system has evolved to improve individual wellbeing by offering a greater choice of provider and care options, as well as faster access for elective treatments [70].

Conceptually, DPH is likely to improve the employer attractiveness of the public sector [14]. Therefore, it would help enhance the access to and quality of healthcare. The increasing number of participants of the FPP service also implies its effectiveness in retaining medical specialists in the public sector. Our review also found that the convenience of performing private practice within the same hospital setting and the attractiveness of government benefits such as special allowances, guaranteed seniority, and promotion with higher salary are the main reasons that specialists prefer to involve in the ‘within’ type of dual practice such as FPP compared to the ‘outside’ form of full private practice [51]. Similar to findings by Ferrinho et al. [4], García-Prado and González [10], and Abera et al. [17], some specialists in Malaysia still decided to stay in public hospitals and join DPH, as they highly value some of the elements in the public sector despite a lower pay. They expressed willingness to devote more efforts and time doing multiple jobs to benefit the ‘best of the two worlds’. Such findings indicated that DPH was effective in retaining public specialists to a certain extent. The review also discussed the growing demand for the FPP Service. Similar to Malaysia, the increasing trend of private patients in public hospitals was also reported in Australia [21] and the United Kingdom [22]. This can be attributed to the enhancement of patients’ choice of medical service delivery within public healthcare by providing alternatives to patients who can afford to pay without compromising the quality of care [23,25]. The freedom to choose an alternative arrangement of amenities such as executive or first class wards, and specialists of preference are among the key features of the dual practice, which makes it appealing to patients [14,25]. This is evidenced by the high satisfaction level reported by the patients under the FPP service [56]. On top of that, DPH improves the efficiency of hospitals, mainly through the optimal resource utilization [32]. However, a high demand for FPP service could exacerbate some of the existing problems in public hospitals, including overcrowding and long waiting time [71]. Waiting time is always used as an indicator of health service quality [72]. Conflicts of interest arising from DPH has also become a concern in Ireland, and the Sláintecare Report [73] called for the termination of DPH due to health inequalities. Another related issue which needs extra attention is the possibility of supplier-induced demand to happen. FPP specialists may perform more procedures or overtreat patients since they are now able to receive more incentives by performing via FPP Service. This may inflate the total healthcare expenditure in Malaysia in the long run. Hence, a further evaluation of the outcomes of the FPP service in Malaysia is warranted.

A portion of the revenue generated from DPH could be reinvested on public hospitals to improve the healthcare quality and alleviate the budgetary pressure [23]. Empirical evidence in certain countries, such as in Rwanda, demonstrated benefits of DPH in the form of upgraded public healthcare facilities and revenue flows [74]. DPH represents as important source of extra revenue for public hospital in Ireland, Australia, and the United Kingdom [14,21,22,23,24]. In fact, the NHS in the UK has encouraged hospitals to generate additional incomes from DPH [22,23]. The UK passed the Health and Social Care Act 2012, which increased the allowed amount to be earned from DPH from 2% to 49% of total hospital earnings [23,75]. Patients seeking subsidized care could also indirectly benefit from the improved healthcare quality [23]. However, as revenues generated from the FPP service is returned to the central treasury in Malaysia [34,54], it is important to ensure that they are used for healthcare. While poor financial planning and management for DPH have been reported [21,22,25], the issue of consumables not listed under the FPP Fee Schedule raised by Amir et al. [51] and Hairusnizan et al. [57] also warrants immediate attention.

Most of the challenges of the FPP Service are associated with the sharing of infrastructure and workforce. Ideally, the FPP service must be delivered effectively without compromising the level of responsiveness to patients seeking subsidized healthcare [13,14]. As argued by Kiwanuka et al. [8], the success of private care embedded within public facilities highly depends on adequate resource availability in terms of infrastructure, resources, and personnel to deliver private care on top of the routine service for public patients. Failing so, it is potentially likely that the resources are shifted to the private care sector within the public facility, whether intentionally or not [46], subsequently leading to the misuse of public resources [10,13] that will compromise the quality of public health services. If these challenges are not addressed, they can corrode public trust towards public service delivery as highlighted by Mueller and Socha-Dietrich [14].

DPH is complicated and reported to add additional burden to hospital administration [69], similar to as highlighted in the literature [14,24,25]. Hospital authorities need to deal with various aspects of DPH, including revenues, service delivery and resource management, if it is adopted. In several countries, hospitals are required to maintain a separate account for DPH to avoid cross-subsidization between public and private care [14]. While health authorities used different ways to cope with the challenges [19], it is important for policymakers to provide them with sufficient training and resources in addition to clear guidelines [23].

This review also highlighted the achievements of the FPP Service in ten public hospitals and suggested DPH expandability to other hospitals in Malaysia. Nevertheless, the concerns about the widening of health inequalities caused by DPH [18,22,73] should be addressed, as its impacts are often context-specific and multifactorial [9].

Undeniably, dual practice is a complex phenomenon with ambiguous effects on the healthcare sector’s performance [10]. In general, some of the criticisms surrounding dual practice was its negative impact on the quality, efficiency, and equity of the delivery of healthcare services [5], especially prevalent in low- and middle- income countries due to weak governance, suboptimal monitoring, and poor supervision [20]. Despite the increasing prominence of dual practice worldwide, there is still a lack of rigorous and scientifically reliable evidence to determine the net impact of this practice [6,10,12,14,20,26]. Therefore, it remains impossible to conclude if the benefits of dual practice outweigh its negative consequences. The achievement of universal healthcare coverage may be impeded by unregulated dual practice [9]. Many unanswered questions revolve around the effects and outcomes of dual practice. Thus, a more in-depth study is needed to analyze the consequences of DPH in Malaysia to facilitate decision makers in strategizing the best interventions to optimize the potentials of dual practice.

Dual practice policy and regulatory response continues to be a topic that generates wide interest among researchers [5,6,7,8,10,11,13,26,46]. González and Cuadrado [7] highlighted that it is still challenging to implement dual practice in most of the low- and middle-income countries due to the lack of regulations. Similarly, Hipgrave and Hort [5] as well as García-Prado and González [10] stated that weak governance and lax regulation of dual practice in developing countries led to a higher possibility of negative consequences. In Malaysia, the FPP service was regulated by limiting the specialists’ engagement, incomes, time spent and the number of patients. Although sharing facilities are likely to ease the governance of the FPP Service [10], the concerns of specialists about the misuse of resources should be addressed. The different viewpoints from the specialists regarding the FPP service monitoring need to be further explored [51]. If DPH is to be expanded in Malaysia, MOH should ensure adequate monitoring capacity for hospitals involved. A strong monitoring structure will ensure a good regulation and the eventual success of dual practice [8].

This article provided an overview of the FPP service as the DPH in the Malaysian public healthcare system using a narrative review approach, also known as “unsystematic narrative review” of relevant sources of data as described in previous publications [49]. This review was limited by the unsystematic literature search method, which potentially caused bias in the literature selection and interpretation [48], despite the critical analysis performed on the literature [49]. Other limitations include the small number of hospitals involved in FPP Service in Malaysia and limited scope of findings from the review.

## 4. Conclusions

As shown by our analysis in Malaysia, the organization and regulation of FPP service is a complex process. Conceptually, DPH such as FPP renders certain benefits but at the same time comes with multiple conundrums. In Malaysia, the FPP Service in ten public hospitals has been well-governed and regulated by the MOH. Though currently there are limited number of FPP hospitals, evidence thus far indicated that it has some potential to retain specialists in public healthcare. It offers additional incomes to specialists and generates extra revenue for government. DPH offers a unique arrangement for public specialists as it enables them to attend to patients in public hospitals, including those who opt out of subsidized healthcare. It is crucial not to overlook the possibility of unequal treatment of public patients with the expansion of DPH in Malaysia so that the vision of universal health coverage will not be derailed. Proactive governance and robust systems of monitoring and regulation are key to ensure the FPP Service in the Malaysia public healthcare sector is sustainable and support patient choice, workforce attraction and retention. Our review also revealed that despite the increasing scale of DPH in Malaysia, there remains a big gap in the relevant literature, especially in the local context. Scientific study and further analysis from different perspectives, for example the impact of this policy on patients’ health and financial implications are warranted to better understand the impact of this type of dual practice in Malaysian public hospitals.

## Figures and Tables

**Table 1:** Profiles of the ten hospitals with FPP service.

Name of Hospital	Type of Hospital	Year of FPP Implementation	Clinical Departments Involved in FPP Service
Putrajaya Hospital	Major specialist hospital	August 2007	General Medicine, Endocrinology, Oral Surgery and Maxillofacial, Obstetrics and Gynecology, Ophthalmology, Orthopedic, Otorhinolaryngology, Pediatric, General Surgery, Endocrine and Breast Surgery, Psychiatry, Rheumatology, Anesthesiology and Intensive Care, Pathology, Radiology.
Selayang Hospital	Major specialist hospital	August 2007	General Medicine, Obstetrics and Gynecology, Ophthalmology, Anesthesiology and Intensive Care, Urology, General Surgery, Hepatobiliary, Radiology, Orthopedic, Otorhinolaryngology, Pathology, Oral Surgery, Hepatology, Pediatric, Nephrology
Sarawak Heart Centre	Special institution	January 2015	Cardiology, Cardiac Anesthesiology and Perfusion, Cardiothoracic Surgery
Queen Elizabeth II Hospital	Major specialist hospital	February 2015	Neurosurgery, Cardiac Anesthesiology and Perfusion, General Surgery, Endocrine and Breast Surgery, Otorhinolaryngology, Orthopedic, Pathology, Radiology, Anesthesiology and Intensive Care, Plastic Surgery, Cardiothoracic Surgery
Sultan Ismail Hospital	Major specialist hospital	June 2015	Oral Surgery and Maxillofacial, General Surgery, Otorhinolaryngology, Obstetrics and Gynecology, Anesthesiology and Intensive Care, Radiology, Orthopedic, Rehab.
Ampang Hospital	Major specialist hospital	June 2015	General Medicine, Otorhinolaryngology, Ophthalmology, Hematology, Oral Surgery and Maxillofacial, Pediatric Dentistry, Radiology, Orthopedic, Obstetrics and Gynecology, Anesthesiology and Intensive Care, General Surgery, Pathology
Sungai Buloh Hospital	Major specialist hospital	September 2015	General Medicine, Neurosurgery, Pediatric, Orthopedic, Obstetrics and Gynecology, Anesthesiology and Intensive Care, Pathology, Radiology, Ophthalmology, Otorhinolaryngology, Pediatric Dentistry, Oral Surgery and Maxillofacial, Rehabilitation Medicine
Pulau Pinang Hospital	State hospital	November 2015	Cardiology, Dermatology, Diagnostic Radiology, General Surgery, Neurology, Obstetrics and Gynecology, Ophthalmology, Otorhinolaryngology, Orthopedic, Pathology, Pediatric, Anesthesiology and Intensive Care, Cardiothoracic Surgery, Urology, Cardiac Anesthesiology and Perfusion
Serdang Hospital	Major specialist hospital	June 2016	Cardiology, Ophthalmology, Anesthesiology and Intensive Care, Cardiothoracic Surgery, Cardiac Anesthesiology and Perfusion, Pathology, Radiology
Sultanah Aminah Hospital	State hospital	November 2016	Obstetrics and Gynecology, General Medicine, Plastic Surgery, Nuclear Medicine, Pathology, Radiology, Anesthesiology and Intensive Care

Reprinted with permission from Medical Development Division [59]. Copyright 2020 Ministry of Health Malaysia.

**Table 2:** Distribution of fees between specialists and federal government revenues.

Type of Fee		Distribution Rates (%) of FPP Service Revenue	
		Specialist Revenue	Government Revenue
Registration fee		-	100
Consultation fee		100	-
Hospitality fee		-	100
Investigation fee		50	50
Procedure fee		100	-
Treatment fee		50	50
Miscellaneous fee			
	Medical check-up packages and other medical packages	60	40
	Medical report	100	-
	Consumables	-	100

Reprinted with permission from Hospital Management Services Unit [30]. Copyright 2015 Ministry of Health Malaysia.

## Data Availability

The data presented in this study are available on request from the corresponding author. The data are not publicly available due to third-party data. Restrictions apply to the availability of these data. Data was obtained from Ministry of Health Malaysia and are available from the authors for researchers who meet the criteria for access to confidential data with permission of Ministry of Health Malaysia.

## Supplementary Materials

The following supporting information can be downloaded at: https://www.mdpi.com/article/10.3390/healthcare10102097/s1. Table S1: Percentage of specialists’ participation in FPP Service in ten FPP hospitals, 2015–2017, Table S2: Percentage of specialists’ resignation from ten FPP hospitals, 2015–2017, Table S3: The trend of specialists’ resignation rate in MOH, Putrajaya Hospital and Selayang Hospital, 2014–2016, Table S4: Total number of non-FPP MOH specialists, FPP MOH specialists, and private facilities’ specialists and percentage of increment by year, 2017–2018, Figure S1: Trend of FPP patients’ encounters, 2008–2019, Table S5: Total number of non-FPP, FPP and private patients’ encounters and percentage of increment by year, 2017–2019, and Table S6: The percentage of FPP patients’ encounters in ten FPP hospitals, 2017–2019.
