# Supplementary material for: Specialists’ Dual Practice within Public Hospital Setting: Evidence from Malaysia
Source: Healthcare (Basel). 2022 Oct 20;10(10):2097. doi: 10.3390/healthcare10102097 (PMC9601889; doi:10.3390/healthcare10102097)
Supplement: Supplementary file 1 [file healthcare-10-02097-s001.zip › healthcare-1853141-supplementary.pdf]

Table S1. Percentage of specialists' participation in FPP Service in ten FPP hospitals, 2015-2017.

Year	Total no. of specialists in 10 hospitals	Total no. of FPP specialists	Percentage (%)
2015	1116	241	21.6
2016	1167	235	27.0
2017	1157	342	29.6

Source: National Audit Department [50].

Table S2. Percentage of specialists' resignation from ten FPP hospitals, 2015-2017.

Year	No. of specialists' resignation		
	Total (MOH)	Total 10 FPP Hospitals	Percentage (%)
2015	165	59	35.8
2016	158	46	29.1
2017	170	52	30.6
Total	493	157	31.8

Source: National Audit Department [50].

Table S3. The trend of specialists' resignation rate in MOH, Putrajaya Hospital and Selayang Hospital, 2014-2016.

**Year**	**2014**		**2015**		**2016**	
	Total number resigned	Resignation rate	Total number resigned	Resignation rate	Total number resigned	Resignation rate
MOH	144	3.6%	164	3.9%	158	3.5%
Putrajaya Hospital	1	1.2%	3	3.5%	2	2.2%
Selayang Hospital	6	3.4%	9	5.1%	10	5.1%

Source: Amir et al. [61].

Table S4. Total number of non-FPP MOH specialists, FPP MOH specialists, and private facilities' specialists and percentage of increment by year, 2017-2018.

Year	Non-FPP MOH specialists		FPP MOH specialists		Private specialists	
	Total No. (excluding FPP specialists)	Percentage of increment by year (%)	Total No.	Percentage of increment by year (%)	Total No.	Percentage of increment by year (%)
2017	6,601	1.7	342	8.6	4,741	6.1
2018	6,461	-2.1	360	5.3	4,865	2.6

Source: Health Planning Division, Ministry of Health Malaysia [31], Fadzil et al. [60].

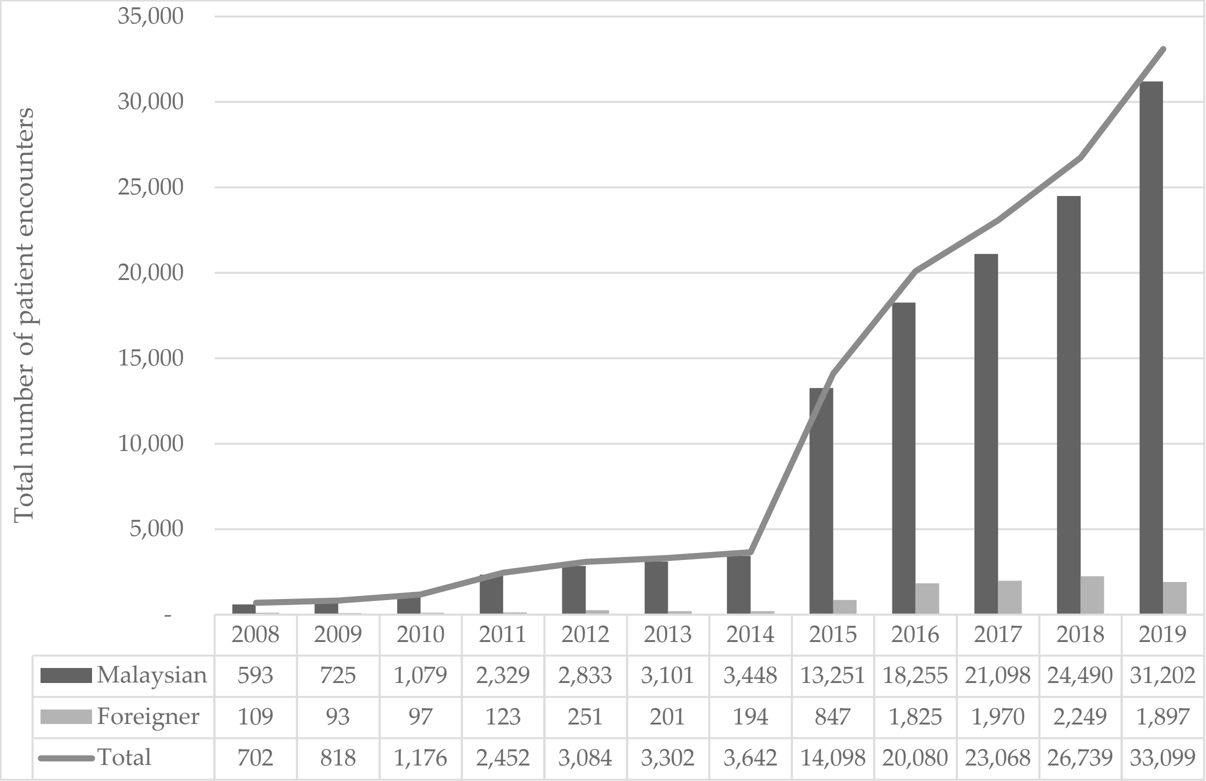

Figure S1: Trend of FPP patients' encounters, 2008-2019. Source: Fadzil et al. [60].

Table S5. Total number of non-FPP, FPP and private patients' encounters and percentage of increment by year, 2017–2019.

Year	Non-FPP patients' encounters at all 146 MOH hospitals (inpatient, outpatient, daycare)		FPP patients' encounters at 10 FPP hospitals (inpatient, outpatient, daycare)		Private patients' encounters at private hospitals (inpatient, outpatient)	
	Total No.	Percentage of increment by year (%)	Total No.	Percentage of increment by year (%)	Total No.	Percentage of increment by year (%)
2017	24,744,818	-1.1	23,068	14.9	4,714,685	-3.7
2018	25,949,686	4.9	26,739	15.9	4,916,437	4.3
2019	26,449,323	1.9	33,099	23.8	4,719,336	-4.0

Source: Ministry of Health Malaysia [38,62-65], Fadzil et al. [60].

Table S6. The percentage of FPP patients' encounters in ten FPP hospitals, 2017-2019.

Year	Total No. of patients' encounters in MOH hospitals (inpatients, outpatients, daycare)	Total No. of FPP patients' encounters in 10 FPP hospitals (inpatients, outpatients, daycare)	Percentage (%) of FPP patients' encounters in 10 FPP hospitals
2017	24,767,886	23,068	0.09
2018	25,976,425	26,739	0.10
2019	26,482,422	33,099	0.13

Source: Ministry of Health Malaysia [38,62-65], Fadzil et al. [60].
